# Mixtures of Gaussian processes for robotic environmental monitoring of emission sources

**DOI:** 10.1007/s10661-025-14059-6

**Published:** 2025-05-06

**Authors:** Ivar-Kristian Waarum, Alouette van Hove, Thomas Røbekk Krogstad, Kai Olav Ellefsen, Ann Elisabeth Albright Blomberg

**Affiliations:** 1 Norwegian Geotechnical Institute, Oslo, Norway; 2 Department of Informatics, University of Oslo, Oslo, Norway; 3 Department of Geosciences, University of Oslo, Oslo, Norway; 4 Norwegian Defence Research Establishment, Kjeller, Norway

**Keywords:** Environmental monitoring, Robotics, Sampling, Gaussian process

## Abstract

Emission of greenhouse gases such as methane and carbon dioxide is a known driver of atmospheric heating. Traditional and emerging industries need innovative solutions to comply with increasingly strict sustainability demands and document environmental impact. Mobile sensor platforms such as aerial or underwater vehicles with a high degree of autonomy present a cost-efficient option for environmental monitoring. Autonomous vehicles commonly use Gaussian processes (GPs) for online statistical modelling of concentrations of environmental features. Emission sources in the monitoring area introduce a complication, since the variance is likely heterogeneous between areas dominated by influx and areas with background concentrations. Mixtures of GPs have previously been demonstrated to be effective in such scenarios. Mixture methods distinguish between the natural background concentration and emission to improve model performance when predicting concentrations and variance at unsampled locations. The mixing of GP models allows for nonstationarity and anisotropy in the modelled spatial dynamics, which is desirable for emission modelling in environments with advective forces such as wind or water current. In this paper, we compare different approaches to spatial concentration modelling that accommodate heterogeneous dynamics, based on mixtures of GPs. Distinction of background and emission is either data-driven or derived from domain knowledge. The predictive performance of different mixture methods is demonstrated on field measurements near emissions and compared in an online path planning context. We identify and discuss important trade-offs between data-driven and knowledge-based clustering of measurements. Results show that mixture methods give realistic variance estimates, suitable for online planning.

## Introduction

Monitoring of chemical, physical, and biological features is one of the most direct methods to gather evidence for assessing the health of our atmosphere, oceans, and forests. In situ monitoring with high spatial resolution enables documentation of the impact that anthropogenic activities have on local ecosystems (Pause et al., 2016). Industries and government agencies need cost-efficient, in situ monitoring solutions combining large area coverage with high frequency observations, both to comply with increasingly strict regulations and for sustainable management of natural resources (Seifert-Dähnn et al., 2021). Autonomous robotic sensor platforms, such as uncrewed aerial vehicles (UAVs) and autonomous underwater vehicles (AUVs), are enabling technologies for cost-efficient data acquisition. With these platforms, it becomes feasible to survey large areas, while sampling densely enough to capture the spatial dynamics of natural phenomena such as dispersion of gas emissions in air or water. A recent overview of robotic gas emission mapping, including different types of sensors and UAV platforms, is given by Francis et al. (2022) and Hossein Motlagh et al. (2023).

Goals for emission-related surveys include source localization, plume mapping, boundary tracking, and flux estimation (Hutchinson et al., 2019). Planning a survey to achieve such goals is challenging if the environment is unknown a priori, for instance the existence and location of emission sources. Similarly, the dispersion of emissions may be affected by wind or water currents, the direction and strength of which are turbulent and difficult to predict (Hossein Motlagh et al., 2023).

If the mobile platform can adapt to an unknown environment by replanning its path during a survey, it can spend more survey time acquiring samples related to survey goals and spend less time acquiring unnecessary samples. Various strategies for adaptive behaviour have emerged, like gradient-based, bio-inspired (gradient-based with fallback search), and information-driven (Sheng et al., 2024). The latter can have better performance than gradient-based methods in turbulent environments (Hutchinson et al., 2019).

Information-driven adaptive path planning is based on a structured and probabilistic representation of the knowledge the robot has of its surroundings—the *belief state*. For emission-related surveys that focus on identification of source location and gas flux, the belief state may a probabilistic distribution model of the source terms (van Hove et al., 2024). For more general mapping surveys, a common way to realize the belief state in autonomous platforms is by using a Gaussian process (GP) model to aggregate sensor measurements. GP models allow prediction of measurement values at locations that have not been sampled, along with a quantification of the predictive uncertainty. GP models are therefore convenient as belief states for environmental monitoring since they in practice constitute a spatial map of measurements, estimations, and uncertainty. In geostatistics, regression or prediction based on GP models is referred to as *kriging*. The theory was developed for the spatial domain by Matheron (1963). A thorough review of kriging can be found in Cressie (1989), and examples of kriging for prediction of density distributions in air include studies on particulate matter (Shukla et al., 2020) and radon gas (Pásztor et al., 2016).

In the context of machine learning, GPs are treated by Rasmussen and Williams (2005). An example where GP models prove useful for path planning is presented in Aniceto dos Santos and Vivaldini (2022), where a GP belief state is integrated with a planning routine that increases the effectiveness of UAV surveys for forest management. In an oceanic environment, Das et al. (2015) demonstrated how a GP could be used for online computation of optimal locations for water sampling with an AUV. Moving on towards time-variant belief state modelling, Berget et al. (2018) used an AUV with a GP proxy model of mass transport to predict and track turbidity plumes of particles suspended in water.

Two recognized shortcomings of GP models are that the computational complexity is cubic with the number of observations and that the covariance function is commonly assumed to be stationary across the domain (Rasmussen and Ghahramani, 2001). The computational complexity raises challenges for use with large data sets and on robotic platforms with limited processing power. Extensive efforts have been made to make GP models more computationally efficient, for instance using low-rank approximations to reduce the complexity (Higdon, 2002; Banerjee et al., 2008) or tailored matrix methods (Gardner et al., 2018). Stationary covariance is problematic when used in an environment where advective forces such as wind or water currents dominate mass transport of, e.g. gas emissions: locations near an emission source can be expected to have a higher spatial variability in gas concentration than locations farther away (Ražnjević et al., 2022). To efficiently model nonstationary environments with fidelity, Tresp (2000) proposed a combination of multiple GP models with different spatial variabilities, weighted to represent different dynamics in the data set. The weights were computed per data point, Other multi-model approaches to model nonstationarity has been proposed by, e.g. Higdon (1998) and Pintea et al. (2018), where the data sets were segregated explicitly in the spatial domain to identify kernel parameters for each model. Also, dimensional warping has been proposed as a method to use stationary models on nonstationary data (Bornn et al., 2012; Sampson and Guttorp, 1992; Vu et al., 2023).

A general overview of the state of the art of combining multiple GPs is given in Liu et al. (2020), where the performance and fidelity of methods such as naive local experts (NLE) and mixture of experts (MoE) are assessed. Approaches to data clustering for mixture methods were presented by van Stein et al. (2020), focusing on reducing the computational complexity of training GP models rather than how different clustering methods can be suitable for particular environments. Chen et al. (2023) introduce a method for data clustering and weighted combination of a predefined set of GP models, i.e. without training the models on the data.

Both Stachniss et al. (2008) and Morales et al. (2022) demonstrate MoE GPs for gas distribution mapping, with the aim that one model represents the low variability of areas with background concentration only, and the other model represents areas with high variability. In both publications, experiments are carried out in controlled environments with a single gas source.

In this paper, we demonstrate and analyze the strengths and weaknesses of different methods of combining or mixing GPs for belief state modelling during environmental monitoring surveys. We base our study on two real data sets with nonstationary spatial correlation structure. Our main contribution is a systematic comparison of data-driven and knowledge-driven approaches to combining GPs, with a performance assessment using real world data sets. The contribution includes an algorithm for knowledge-driven clustering of data in a 2D spatial setting. Furthermore, we discuss the performance of different approaches to data clustering both in the environmental monitoring context and the adaptive path planning context.

Three approaches to GP modelling are presented in the following section. The approaches are tailored for robotic environmental monitoring of areas where one or more emission sources are potentially present, and the advective forces dominate the diffusion rate. Parts of the measurement series are classified as either emission related (from a source) or background concentration, and the region pertaining to each part is handled differently in the modelling. The presented approaches differ in how they classify measurements: The MoE GP employs a data-driven approach similar to Stachniss et al. (2008), where the measurements are classified as part of the training procedure. In contrast, the NLE GP we present here is a novel approach for gas distribution mapping, where domain knowledge is used for data clustering. The third approach is a modification of NLE, where model averaging (NLE-MA) is used to avoid sharp transitions in the predictions between locations with background concentration and locations with emission.

In the “Results” section, we demonstrate and compare the performance of the tailored modelling approaches on the two data sets, along with the performance of a standard GP model. The comparison is done by predicting the ‘true’ scalar field for each scenario, including the predictive variance. We systematically compare predictions produced by the variants of GPs and assess the impact of different clustering methods used on realistic measurements of gas emission. The paper proceeds with a discussion of pros and cons of the different approaches in light of the typical goals for environmental monitoring such as mapping extent of emissions and source localization, before some concluding remarks in the final chapter. Finally, we evaluate the suitability of the different variants of GPs in the robotic environmental monitoring context.

## Methods and data sets

Whether a robotic sensor platform is following a pre-programmed set of waypoints or using an adaptive sampling algorithm to compute a path in real time, the aim is to acquire samples that capture the spatial variability in the area. This data set constitutes a sparse subset of the true scalar field. Hyperparameters of the GP—or mixture GP—model are then trained on the data set in order for the model to be able to predict the true scalar field.

This section first introduces our spatial setting and the GP framework, followed by the different variants of mixture GP modelling which allows building augmented models that can accommodate eventual nonstationarity. Then, the two data sets with gas concentration measurements from different locations are presented.

In order to be relevant for practical application with autonomous vehicles, the methods must have an acceptable computational load to run on the vehicle. Since the data sets here are already acquired, we emulate the acquisition by separating each data set into subsets for training and test. The first parts of the Barter Island and Tjøtta data sets are used to train GP models for each location. Then, the localized models are used for modelling and prediction together with the latter parts of the data sets. The same approach is practical in an online setting, where a survey can be carried out in two stages. In the first stage, the vehicle samples the environment until some target distance is covered, or a time limit is reached, before training GP models on the acquired data. In the second stage, the vehicle employs the data and models to predict the emission at unsampled locations and uses the predictions as basis for online path planning. A similar two-stage approach is employed in Fossum et al. (2019). It is worth noting the difference in motivations for using the training/test setup in a robotic sampling context, as opposed to using it in general machine learning. In the latter, the aim is learn something that can be applied in a general setting. In the context presented in this paper, the learning should enable the robot to adapt to a particular environment. For several reasons, and if onboard computational resources allow, it is beneficial to repeat the training regularly based on the most recent data. Retraining is relevant if a survey takes a long time, if the distance covered is large, or if measurements indicate that the environment may have changed (e.g. stronger winds). Time-dependent environments and the retraining aspect are not considered in this paper.

The algorithms herein were implemented mainly using the GPyTorch library, which is state of the art for efficient computations of GPs (Gardner et al., 2018; Wang et al., 2019).

### GP modelling

The GP framework can be used to model both spatial and temporal dependencies between data points. Our data sets were gathered by a vehicle moving through environments, and it is likely that disturbances like changing wind direction are present in the data. In a time-invariant (synoptic) setting, one can assume that there is a series of measurements

1 $$\begin{aligned} \varvec{y} = f(\varvec{X}) + \epsilon , \end{aligned}$$

where $\epsilon$ is a vector of normally distributed measurement noise $\mathcal {N}(0, \sigma ^2_n)$, and *f*() is a mapping between locations $\varvec{X}$ and the measured values $\varvec{y}$. For the spatial applications herein, we will use $\varvec{s} = \{\varvec{X}, \varvec{y}\}:= \{\varvec{x}^T_i, y_i\}^N_{i=1}$ to denote a data set of *N* samples where $\varvec{x} \in \mathbb {R}^2$ represents the location of a sample and $y \in \mathbb {R}$ is the sampled value. $\varvec{s}$ thus represents a scalar field of measured gas concentrations.

A GP is often defined as a collection of random variables where any finite number of variables have a joint Gaussian distribution. Following the definition in Rasmussen and Williams (2005), the GP prior is completely specified by its mean function, $\mu (\textbf{x})$, and covariance kernel function, $k(\textbf{x}, \textbf{x}')$:

2 $$\begin{aligned} f(\textbf{x}) \sim \mathcal{G}\mathcal{P}(\mu (\textbf{x}), k(\textbf{x}, \textbf{x}')). \end{aligned}$$

GP models are commonly said to be nonparametric, meaning that they are not described using a set of model-specific parameters such as linear regression models or models based on physical laws. Instead, a GP defines a prior over the space of a latent function to accommodate any set of prediction points. In Eq. 2, the latent function $f(\textbf{x})$ is defined by a mean function $\mu (\textbf{x})$ and a covariance function $k(\textbf{x}, \textbf{x}')$—where $\textbf{x}$ and $\textbf{x}'$ are two different locations. The covariance function makes it possible to estimate $f(\textbf{x})$ at locations where no measurement has been taken. The predictive distribution at a new point $\textbf{x}_*$ is given by

3 $$\begin{aligned} f(\textbf{x}_*) | \textbf{x}, \textbf{y}, \textbf{x}_* \sim \mathcal {N}(\bar{f}_*, \text {var}(f_*)), \end{aligned}$$

where the kriging equations from Rasmussen and Williams (2005) are

4 $$\begin{aligned} \bar{f}_*= &  \textbf{k}_*^T(\textbf{K} + \sigma _n^2\textbf{I})^{-1}\textbf{y} \nonumber \\ \text {var}(f_*)= &  k(\textbf{x}_*, \textbf{x}_*) - \textbf{k}_*^T(\textbf{K} + \sigma _n^2\textbf{I})^{-1}\textbf{k}_*. \end{aligned}$$

In Eq. 4, $\textbf{K}$ is the covariance matrix containing the covariance functions $\varvec{k}(\varvec{x}, \varvec{x}')$ between the observed locations in the data set $\varvec{s}$, $\textbf{k}_*$ represents the covariances between the observed locations and the location $\varvec{x}_*$ to be estimated, $\varvec{y}$ is the vector of measured values, and $\sigma _n^2$ is the noise variance. A range of covariance kernel families exist, and the choice of which to use is important for how well the GP is able to approximate the underlying scalar field (Rasmussen and Williams, 2005; Duvenaud, 2014). For spatial applications such as gas distribution mapping, the Matérn kernel is often favoured because of its ability to model scalar fields with relatively low smoothness (Matérn, 1966; Stachniss et al., 2009). The Matérn covariance function is parametrized as

5 $$\begin{aligned} k_{\text {Matern}}(r) = \sigma _f^2 \frac{2^{1-\nu }}{\Gamma (\nu )}\left( \frac{\sqrt{2\nu }r}{\varvec{l}}\right) ^\nu K_\nu \left( \frac{\sqrt{2\nu }r}{\varvec{l}}\right) , \end{aligned}$$

where $r = \Vert \textbf{x} - \textbf{x}'\Vert$ is the Euclidean distance between the input points $\textbf{x}$ and $\textbf{x}'$, $\sigma _f^2$ is the signal variance, $\nu$ is the smoothness parameter controlling the differentiability of the function, $\varvec{l} = [l_1, l_2]$ are the length scales, $\Gamma$ is the gamma function, and $K_\nu$ is the modified Bessel function of the second kind of order $\nu$. Commonly for spatial regression, the Matérn covariance function is implemented so that locations that are farther apart have lower covariance. Common choices for the smoothness parameter $\nu$ are 3/2 or 5/2, which makes the covariance function once or twice differentiable respectively. Other choices, like $\nu = 1/2$ and $\nu \rightarrow \infty$, correspond to the exponential and the squared exponential covariance functions.

When GP models are said to be nonparametric, it means that they do not assume a fixed form or a specific number of parameters for the underlying function they are modelling. However, as shown in Eqs. 4 and 5, the kriging model and the covariance function *k* have parameters—sometimes referred to as hyperparameters—and it is the shape of the kernel and the values of these parameters that determine the correlation structure between data points. Identifying the parameter values is done during the training stage of a survey. Following Rasmussen and Williams (2005), the trainable parameters $l_1, l_2, \sigma ^2_n$ can be inferred by maximizing the log likelihood of the observations, given by

6 $$\begin{aligned} \log p(\textbf{y} | \textbf{X}, \theta )= &  -\frac{1}{2} \textbf{y}^T (\varvec{K} + \sigma _n^2 I)^{-1} \textbf{y}\nonumber \\ &  - \frac{1}{2} \log |\varvec{K} + \sigma _n^2 I| - \frac{N}{2} \log 2\pi \,. \end{aligned}$$

Even though the Matérn kernel is flexible and can model relatively sharp gradients of the underlying scalar field, the spatial correlations between locations depend only on their relative distance *r*, as seen in Eq. 5. Some anisotropy can be modelled through the application of two correlation lengths $[l_1, l_2]$, where each correlation length can model a spatial dimension. Hence, if the coordinate system is rotated so that one of the axes coincides with the dominating direction of the advective forces, the correlation structure can take on an elliptic shape instead of being circular. This shape will improve predictions of gas concentration in a windy environment. However, this approach can not accurately capture unidirectional trends such as wind, since the length scales do not distinguish between upwind or downwind directions.

### Mixture modelling

When a GP is used to predict $f(\varvec{x})$, it is implicitly assumed that there is an underlying ‘true’ function that can be realized as a sum of Gaussian distributed variables. Mixing of GPs is a technique that tries to accommodate situations where the spatial dynamics of the phenomenon are nonstationary, or in general can be better modelled through the combination of several GPs. Mixtures of GPs are not in themselves Gaussian, but have more flexible distributions that can handle nonstationarity and multi-modalities in data.

**Table 1:** Variants of mixture models and handling of data clusters

	Identification	Training	Prediction	Complexity
MoE	Implicit	Soft or hard	Soft	$O(3MN^3)$
NLE	Explicit	Hard	Hard	$O(N^3/M^2)$
NLE-MA	Explicit	Hard	Soft	$O(N^3/M^2 + N)$
Single$^{1}$	−	−	−	$O(N^3)$

$^{1}$A single kernel GP as presented in Eqs. 2–4.

Implicit and explicit identification refers to the method of clustering and assigning data points to the mixture GPs. The terms soft or hard indicate whether data points are pertaining to one GP only, or if they can be shared between models

Motivated by the expected variability in spatial correlation, we want to differentiate between modelling and prediction based on data clusters that likely contain an anomaly and modelling and prediction based on clusters with background concentration only. A comparison of different cluster-based methods for modelling and prediction is presented by van Stein et al. (2020). They compare selected methods on a number of data sets and conclude that the two methods based on GP mixture models and regression tree, respectively, have the best overall performance. In common for the two methods is that they cluster the data points based on all the information in the observations $\varvec{s} = \{\varvec{X}, \varvec{y}\}$, as opposed to using only the locations $\varvec{X}$. However, the mixture model and the regression tree methods differ in how clusters are separated. The regression tree method has a hard separation between the clusters, which means that each observation belongs to only one model. Thus, in the training stage, the kernel hyperparameters of the two models are inferred based on different data subsets. The hard separation is retained for prediction, where only one kernel is used, depending on the location to predict. In contrast, the GP mixture method trains kernels simultaneously on all observations and employs all models during the prediction step, where their respective contributions are combined.

In summary, the general approach to modelling with data clusters has three steps:

1. Identify data clusters with anomalies
2. Train and assign GP models to the clusters
3. Predict the gas distribution for unobserved locations

For each step, there are multiple design choices to be made, which distinguishes different approaches to mixture modelling. An overview of design choices is given in Liu et al. (2020), where the distinction between MoE and NLE approaches is explained in detail. In addition to these approaches, we include the NLE-MA variant where model averaging is applied to smooth out the predictions from the NLE models. Table 1 lists the compared methods, along with the main differences in identification of clusters, training, and prediction. It is worth to note that even though we sometimes refer to these models collectively as ‘mixture models’, the NLE method with hard segregation of data points both for training and prediction is not actually a mixture model, since only one GP is used to model each region. A single kernel GP is included as a baseline. The general classification terms implicit/explicit and soft/hard are taken from Liu et al. (2020).

### MoE GPs

MoE GP models can be expressed as an extension of Eq. 2;

7 $$\begin{aligned} g(\varvec{x}) \sim \sum _{m=1}^{M} \pi _m(\varvec{x}) \cdot f_m(\varvec{x}) \end{aligned}$$

where $m=1, \ldots , M$ denotes the different experts, and the gating functions $\pi _m(\varvec{x})$ determine the contribution of each GP to the overall mixture model at each location $\varvec{x}$. The number of experts is a crucial parameter that determines the flexibility of the model, with more experts enabling the capture of increasingly complex and heterogeneous patterns in the data. For gas distribution mapping, the model should be able to reflect the different correlation structures in the background concentration and emission anomalies. Stachniss et al. (2008) demonstrated that using two experts is sufficient to model gas distributions near an emission source accurately. The applications presented herein are likely to have multiple emission sources, but homogenous in nature. Therefore, two experts should be adequate also in our cases. For more complex cases, frameworks exist for mixtures of an arbitrary number of experts (Rasmussen and Ghahramani, 2001; Xu et al., 2020).

An important difference between MoE and NLE methods is that for MoE models, the identification of data clusters is implicit, driven by the data. In addition to inferring the kernel parameters for the models, the aim of the training procedure is now also to infer a responsibility matrix that maps each data point to the different models with a weight corresponding to how important that model is to predict that particular point. When training the MoE, all training data is used to train each model in parallel, but the responsibility matrix is used to weigh the data points during optimization. Tresp (2000) proposed to use an algorithm commonly referred to as the expectation-maximization (EM) algorithm as a way to handle both inferring kernel parameters and assigning responsibilities. The name refers to the two steps in the algorithm: The E-Step calculates the responsibilities $\gamma (s_{im})$, which represents the probability that, based on current parameter estimates, the $i$-th data point belongs to the $m$-th Gaussian component:

8 $$\begin{aligned} \gamma (s_{im}) := p(f_m|\varvec{x}_i, y_i) = \frac{p(y_i|f_m)}{\sum ^M_{j=1} p(y_i|f_{j})}\,, \end{aligned}$$

where $p(y_i|f_m)$ is the log likelihood of doing observation $y_i$ under model $f_m$.

Then, the M-step employs the conjugate gradient descent method to infer parameter estimates for each model that maximize the marginal log likelihoods. The likelihood function is the same as in Eq. 6, except that the noise variance is now given as a diagonal matrix where the variance of each data point is scaled with the corresponding responsibility computed in the E-step:

9 $$\begin{aligned} \varvec{\psi }_m= &  \text {diag} \left( \frac{\sigma _{n, m}^2}{\gamma (\textbf{s}_m)} \right) , \nonumber \\ \log p(\textbf{y} | \textbf{X}, f_m)= &  -\frac{1}{2} \textbf{y}^T (\varvec{K}_m + \varvec{\psi }_m)^{-1} \textbf{y} \nonumber \\ &  - \frac{1}{2} \log |\varvec{K}_m + \varvec{\psi }_m| - \frac{N}{2} \log 2\pi .\nonumber \\ \end{aligned}$$

The EM algorithm continues to iterate the E- and M-steps, gradually computing new responsibilities $\gamma (s_m)$ for soft clustering of the data into mixture components, and converging on estimates for the hyperparameters of these components. Finally, the predictive distribution at a new point $\textbf{x}_*$ is given by

10 $$\begin{aligned} g(\varvec{x}_*) | \textbf{X}, \textbf{y}, \textbf{x}_* \sim \mathcal {N}(\bar{g}_*, \text {var}(g_*)), \end{aligned}$$

where the kriging equations are derived by applying the gating function $\pi _m$ on the predictive means and variances $(\bar{f}_*, \text {var}(f_*))$ for each model *m*:

11 $$\begin{aligned} \bar{g}_* :=&\sum _{m=1}^{M} \pi _m(\varvec{x}_*) \cdot \bar{f_m}(\varvec{x}_*) \nonumber \\ \text {var}(g_*) := &\sum _{m=1}^{M} (\text {var}(f_m(\varvec{x}_*) + (\bar{f_m} - \bar{g}_*))^2 \cdot \pi _m(\varvec{x}_*). \end{aligned}$$

The gating function $\pi _m(\varvec{x}_*)$ reflects the probability that model *m* should be responsible for prediction at location $\varvec{x}_*$. The gating function is used to weigh the influence of different GP models in the output space, in effect smoothing the overall prediction by combining predictions from the different GPs. Since the gating function should be defined in the entire output space, not only for observed locations, we define a gating function by training a gating GP on the final responsibilities $\gamma (s_{m})$ from the EM algorithm. Predictions from the gating GP are then used to estimate the gating function at unsampled locations.

### NLE and NLE-MA

**Fig. 1:**
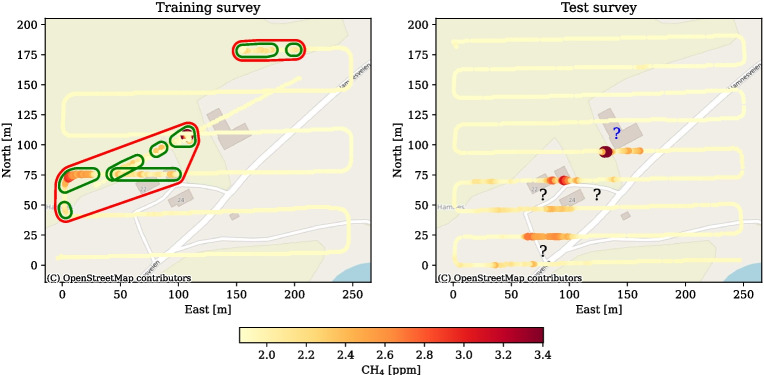
Methane concentration measured over an agricultural plot at Tjøtta, Norway (65.8$^\circ$ N 12.4$^\circ$ E). Both colour and size of markers indicate concentration of CH$_{4}$. The training data set (left) was acquired in two stages: First, the UAV flew over the area of interest along a coarse grid pattern. In the second stage, the UAV flew towards north-east, against the dominant wind direction. The starting position was selected so that the UAV would pass over a suspected emission source: The cattle housed in the L-shaped barn building near the centre of the survey area, marked with a question mark. The measurements have been classified by Algorithm 1, first as clusters of consecutive anomalous measurements (green) and then as emission regions (red). The test data (right) was acquired while doing nine east-west passes over the survey area, with $\mathtt {\sim }24$ m between each pass

Similar to the regression tree from van Stein et al. (2020), the NLE method employs a hard data clustering based on a threshold value on the measured concentration *y*. In the 2D spatial setting, Algorithm 1 uses the observed locations and the threshold value to define anomaly regions in $\mathbb {R}^2$, such as the regions defined by green borders in Fig. 1. The parts of the area of interest that are not inside the anomaly regions constitute the background region. For our purposes, we assume that the anomalies are homogenous, and the scalar field $f_r(\varvec{x}_r)$ within the anomaly regions can be modelled by a single GP, based on the observations $\varvec{s}_r \subset \varvec{s}$ from those regions. The two NLE GPs can therefore be expressed as an extension of Eq. 2 as

12 $$\begin{aligned} f_r(\varvec{x}) \sim \mathcal{G}\mathcal{P}_r(\varvec{\mu }_r(\varvec{x}_r),k_r(\varvec{x}_r,\varvec{x}_r'))\,. \end{aligned}$$

We use the subscript *r* to emphasize the direct link between the GP model and the spatial regions, as opposed to the subscript *m* that was used for the data-driven MoE method. The anomaly and background GPs are trained on observations $s_r$ from their corresponding regions in the training survey. For prediction, it is a design choice whether to inform the model of observations from the other region. In our case, we give the entire set of test observations to the NLE GPs for prediction, to avoid artificially high variance near the region borders. The kriging equations for NLE are as shown in Eq. 4, but care is taken to use the anomaly GP for prediction inside the anomaly region, and the background GP everywhere else.

The NLE-MA method employs the same explicit identification of regions as the NLE method, which leads to the models being trained on different subsets of observations. A drawback with the discrete separation of regions in the NLE method is that discontinuities in the predictive mean and variance are likely to appear along borders between regions. Also, the anomaly regions may include patches of background concentrations, where the anomaly model fits poorly. As a remedy, the NLE-MA method employs both models during prediction, regardless of whether the location to predict is inside an anomaly region or not. A weighted smoothing of the background and anomaly GP predictions is subsequently applied. The model can therefore be expressed as

13 $$\begin{aligned} h(\varvec{x}) \sim \sum _{r=1}^{R} \pi _r(\varvec{x}) \cdot f_r(\varvec{x})\,, \end{aligned}$$

where $\pi _r$ is a gating function and $f_r$ corresponds to the NLE GPs from Eq. 12. Analogous to the MoE method, the gating function $\pi _r$ is another GP trained on the posterior probabilities of the different NLE models at each observed location $\varvec{x}$:

14 $$\begin{aligned} \pi _r := p(f_r|\varvec{x}, \varvec{y}) = \frac{p(\varvec{y}|f_r)}{\sum ^R_{j=1} p(\varvec{y}|f_j)}\,, \end{aligned}$$

where *R* is the number of NLE models. Then, the predictive mean and variance of the NLE-MA mixture model can be computed with Eq. 11.

#### Explicit identification of data clusters and regions for NLE

Selection of threshold values or other ways of manually defining clusters and regions gives explicit control of how the different GPs are trained, and which parts of the observation data set are used for prediction in each region. Expert users can take advantage of this to apply domain knowledge such as which concentration levels are likely to indicate emission and which are likely to appear under natural background fluctuations.

For the drone-based environmental monitoring applications presented herein, the samples in $\varvec{s} = \{\varvec{x}, y\}^N_i$ are taken along the flight path, so that samples $s_{1}$ and $s_{N}$ are at the very beginning and end of the path, respectively. This can be exploited to initiate the spatial clustering of samples. Each sample $s_i$ is classified as representing either an anomaly or background emission. The classification criterion is simply a threshold value on the rolling distance mean or median of the observed concentrations along the flight path. Since the clusters are along-flight only, they contain limited information about the spatial extent of the anomalies. It is desirable to merge clusters so that they can be analyzed together to recover as much information regarding the spatial variation as possible. Therefore, clusters that are sufficiently close to each other are merged into *regions* by drawing a line around neighbouring clusters and adding an arbitrary boundary on the exterior. The resulting regions do not have to be convex.

Algorithm 1 details the procedure of region identification. First, it loops through the samples in $\varvec{s}$ to form a number of clusters $\varvec{C}$ based on the measured concentration along the flight path. Then, the shortest distances between all clusters in $\varvec{C}$ are computed and stored in the distance matrix $\varvec{Z}$. Finally, clusters are merged to regions $\varvec{R}$ based on $\varvec{Z}$ and the distance criterion *d*. The merging procedure utilizes the ‘Density-Based Spatial Clustering of Applications with Noise’ (DBSCAN) algorithm as implemented in scikit-learn (Ester et al., 1996; Pedregosa et al., 2011). In Fig. 1 (left), two anomaly regions are identified.

The spatial extent of the regions in $\varvec{R}$ is used for multiple purposes in the NLE method. For instance, training data for NLE models is identified as $\varvec{s}_r = \{\varvec{X}_r, \varvec{y}_r\} \subset \varvec{s}$, which are the observations pertaining to the anomaly regions. Also, identifying whether new prediction locations $x_*$ are inside or outside the anomaly regions determines if the GP trained on anomalous data or the GP trained on background data should be used for prediction.

**Algorithm 1:**
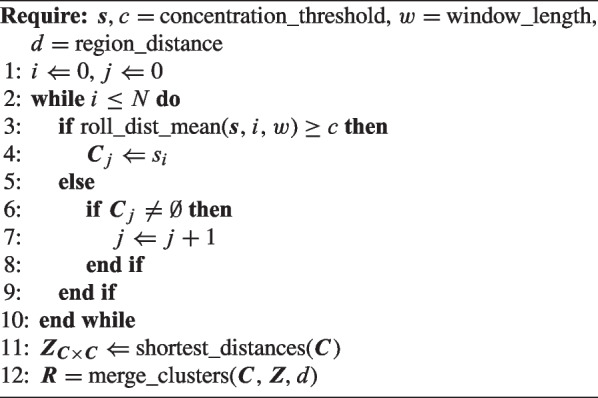
Identify regions of anomaly observations in data set $\varvec{s} = \{s\}^N_{i=1}$.

Algorithm 1 has three free parameters: the concentration threshold *c*, the rolling distance window *w*, and the distance criterion *d* for whether to merge clusters into regions. The concentration threshold divides the data set in two parts: samples indicating an anomaly and samples from the background concentration. The threshold should be set based on a consideration of which concentration levels are considered anomalous—or likely to have a nearby source—and which levels are within the natural background variations. Setting this threshold is therefore based on assumptions about the environment and the constituents in the emission.

In contrast, deciding the distance criterion for merging nearby clusters relates to a consideration of the monitored area and knowledge of potential emission sources. The criterion for merging should partly depend on some initial belief of how likely it is that more than one emission source is present in the area; if all emission is likely to have a single source, clusters relatively far apart can be merged. In addition, the distance criterion should depend on the density of sampling locations. Particularly, if the samples are taken while the sensor platform is following a pattern, the distance should allow merging sample locations from neighbouring grid lines into the same region, since there are no information about the concentration level between the grid lines.

When all anomaly regions $\varvec{R}$ have been identified, the region of the set of samples that represent the background concentration can be defined as $\varvec{b} = \varvec{s} \setminus \varvec{\bigcup }_{i} r_i$.

### Assessing predictive performance

The predictive performances of the GP methods presented here are assessed quantitatively using a number of metrics that examines the predictive means and the predictive variance. The RMSE is computed between the observations and the model predictions at the observed locations:

15 $$\begin{aligned} \text {RMSE} = \sqrt{ \frac{1}{N} \sum _{i=1}^{N} \left( y_i - y_{*i} \right) ^2 }\,. \end{aligned}$$

The empirical interval coverage (EIC) describes how well the predicted confidence intervals matches the actual variability in the data and is computed as

16 $$\begin{aligned} \text {EIC}_{\alpha } = \frac{1}{N} \sum _{i=1}^{N} \textbf{1} \left[ y_i \in \left[ \mu _i - z_{\alpha } \sigma _i,\ \mu _i + z_{\alpha } \sigma _i \right] \right] \,, \end{aligned}$$

where $\sigma = \sigma _n + \sigma _f$ is the model standard deviation and $\textbf{1}$ is an indicator function that equals 1 if the condition is true, 0 otherwise, and $\alpha$ is the confidence interval, e.g. $z_\alpha = 1.96$ for $\alpha = 95\%$.

Finally, the mean continuous ranked probability score (CRPS) is computed with a Monte Carlo sampling from the model predictive distributions, as

17 $$\begin{aligned} \text {CRPS}(\varvec{y}_*, \varvec{y})\approx &  \frac{1}{K} \sum _{k=1}^{K} \left| \varvec{y}_{*k} - \varvec{y} \right| \nonumber \\ &  - \frac{1}{2K^2} \sum _{k=1}^{K} \sum _{l=1}^{K} \left| \varvec{y}_{*k} - \varvec{y}_{*l} \right| \,, \end{aligned}$$

where *K* is the number of sampling rounds, and $\varvec{y}_{*k}, \varvec{y}_{*l}$ are samples of the predictive distributions at the location of all observations $\varvec{y}$. The first term is the average distance from the samples to the observed values, while the second term penalizes large pairwise distance between samples. The Monte Carlo approach is used because the closed form CRPS is valid only for unimodal GPs. The CRPS is used here as a metric that scores both the mean and variance of the predictive distribution: It rewards narrow distributions if they are near the observed value, and broader distributions if they are needed to represent the truth due to uncertainty.

**Fig. 2:**
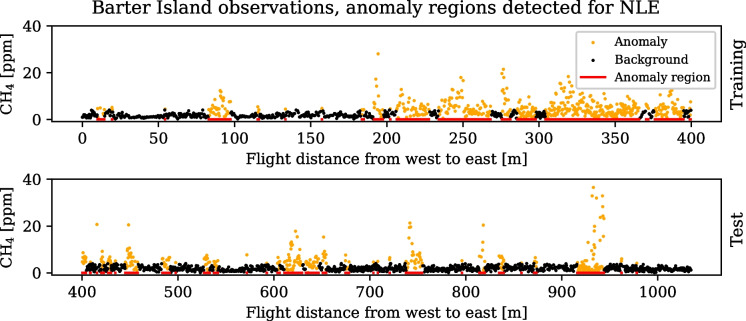
Methane concentration measured along 2 km of coastline on the north side of Barter Island, Alaska, USA, in 2018. The measurements have been classified for NLE modelling, as either related to an anomaly (orange) or the background concentration (black)

### 1D data set from Barter Island

We apply the presented methods for data clustering and prediction to two data sets that constitute field data gathered in situ in natural, uncontrolled environments subject to environmental noise, wind disturbance, and sensor imperfections. The first data set is a publicly available measurement series from a methane survey over thawing permafrost in the arctic (Oberle, 2019). The second data set is from a methane survey above an agricultural plot in northern Norway in 2023. The data sets differ in dimensionality; the observations in the first data set were collected along an almost linear transect, while the second set of observations represent spatially distributed locations in both horizontal dimensions.

A 1D case, where observations are taken along a straight line in space, allows us to compare some fundamental aspects of the different approaches to mixture GP models. Importantly, the data series should cover sections with different spatial variance, so that the transitions between sections can be studied. The data set from Barter Island shows the variation of methane concentration in the air over soil with thawing permafrost. The data is thoroughly presented in Oberle et al. (2019) and is available online under the CC-BY-4.0 licence (Oberle, 2019). A UAV equipped with a methane sensor surveyed along a coastline, measuring the concentration of methane over a distance of 2000 m. The flight path traversed several regions with relatively high methane concentrations. For demonstration purposes, this paper uses the first half of the data set, totalling 2570 observations over 1050 m as shown in Fig. 2. The UAV flew in a straight line from west to east; therefore, the transverse spatial dimension is ignored here.

To prepare the measurements for multi-kernel modelling, subsets of the distance are separated based on a running average of the median value of a 4-m long window. The aim is to separate between background concentration and anomalous measurements, similar to what Algorithm 1 does in the 2D case. Regions where the median is above the chosen threshold are considered to represent an anomaly. All other locations are considered to represent the background concentration of methane. Figure 2 shows the separation into anomaly and background measurements, where the concentration threshold was determined through a combination of domain knowledge, in situ observations, and sensor performance.

When all anomalous measurements are gathered into one data set, there is an implicit assumption that the anomalies have similar dynamics and one model can be trained to represent all areas with anomalies. For the Barter Island data, this assumption can be defended since the increased concentrations can be attributed to the same phenomenon: waterways transporting dissolved methane, that is subsequently fluctuating into the air (Oberle et al., 2019). In other cases, for instance a survey of an area with multiple sources that have different types of origins, it would be beneficial to distinguish between subsets of anomalous measurements.

### 2D data set from Tjøtta Island

We further demonstrate the potential of different approaches to mixture GP models for environmental monitoring on a 2D data set of observations collected along a grid survey. In particular, we demonstrate how mixture GPs can accommodate nonstationarity and anisotropy in the observations. These effects are present in observations of gas concentrations in a windy environment.

We apply the GP mixture models to atmospheric methane observations from an agricultural site in Tjøtta, Norway. The training and test surveys took place on consecutive days in August 2023, with similar environmental conditions. Throughout the survey, wind was north-easterly at approximately 1.5 m/s. A DJI Matrice 300 RTK drone equipped with an Aeries MIRA Strato LDS methane sensor was used to survey gas emission over a cattle farm. Flight paths and observations are shown in Fig. 1. Flying height was $\sim$25 m, above the tallest ventilation chimney on the barn. The UAV travelled at a horizontal velocity of 1.8 m/s, and the sensor obtained methane concentrations at a sampling rate of 1 Hz. In total, 2570 and 1257 observations were acquired for the training and test data sets, respectively. The data sets are available online under the Creative Commons Attribution licence (van Hove and Pirk, 2024).

## Results

The applicability of the MoE, NLE, and NLE-MA modelling methods is demonstrated for modelling emissions in an environment with directional forces such as wind or water currents. The data sets from Barter Island and Tjøtta differ in dimensionality, and each case needs some special consideration for training and prediction. The three models are trained and used for prediction for both data sets, enabling us to compare predictive means, variances, and running times.

### Barter Island

The training and test subsets of observations from Barter Island are shown in Fig. 2. The initial 400 m of observations were used as training data.

#### MoE in 1D

Training of the MoE GP was done with the EM algorithm as summarized in Eqs. 8–9. In the E-step, the likelihoods of each observation belonging to each model were computed and then normalized so that the probabilities of a point belonging to each model sums to one. During the M-step, gradient descent optimization was used to find hyperparameters for each model, using the observations that were most likely to belong to that model. We follow the implementation of the M-step in Stachniss et al. (2008), where the assignment of observations during optimization was done by adding noise proportional to the likelihood. Since all likelihoods are equal before training of hyperparameters starts, the observations with the highest $10\%$ of concentrations were assigned to one of the models as initialization.

#### NLE and NLE-MA in 1D

As an alternative to the MoE model trained with the EM algorithm, where the anomaly and background observation subsets are identified during training, the observation subsets are for the NLE methods based on domain knowledge. As an example of domain knowledge, the NOAA Global Monitoring Laboratory regularly reports the trend of atmospheric CH$_{4}$, which in September 2017 was $\mathtt {\sim }1.85$ ppm (Lan et al., 2024). A threshold that separates anomaly observations from the background can then be set based on this. Incorporating site-specific knowledge may further improve the ability to set meaningful parameters for the training of GPs. For the Barter Island data series, shown in Fig. 2, Oberle et al. (2019) reports an instrument variance of 1.17 around a linear average of 2.06 ppm during a test flight in a location where there should be no sources of emission. The variance thus corresponds to $\pm 56\%$ of the measured signal in that particular field setting, which exceeds the error range of $\pm 10\%$ promised by the sensor manufacturer. Assuming that the extra $\mathtt {\sim }46$% in variance was due to environmental disturbances, e.g. wind-dispersed gas, we limited the likelihood noise to $\sigma _n \le 1.0$. The threshold for anomaly classification was set to 4.2, which corresponds to the 95% confidence interval of the test flight, assuming normal distribution around the mean.

**Fig. 3:**
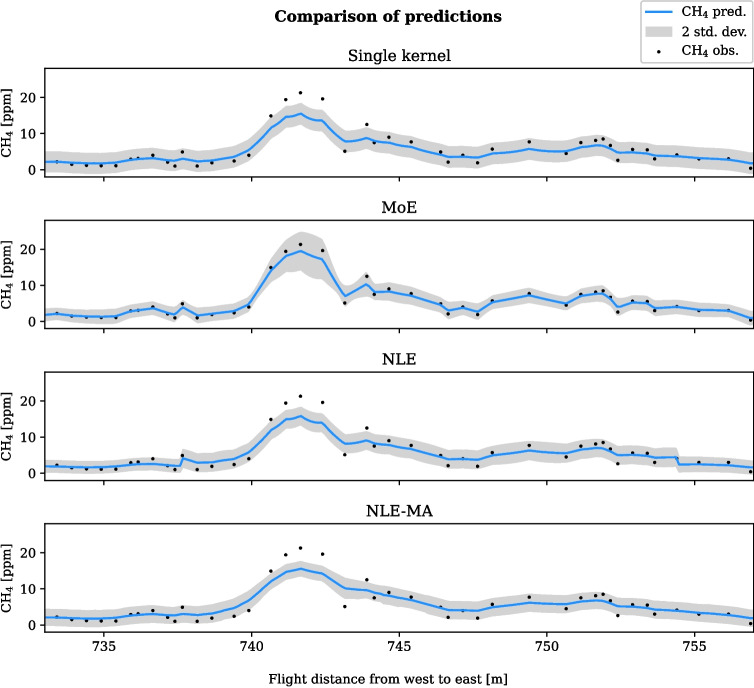
Predictive mean and two standard deviations for a subset of the flight path are shown for three different mixture models, along with a single kernel model as baseline. Most of the CH$_{4}$ observations from the stretch between 737 to 754 m are above the set threshold for the NLE methods, and the section is therefore modelled as an anomaly

#### Comparison of methods—1D

The test subset of observations was defined to be from 400 to 1050 m along the flight path. In order to evaluate the performance of different models, we inspect a shorter stretch of the path that exhibits some details on anomaly and background predictions. Figure 3 shows the predicted mean and variance for the NLE, NLE-MA, and MoE models, respectively, along with predictions from a single kernel as baseline. Between 735 and 755 m along the flight line, a stretch with anomalously high concentrations was encountered.

The predictive mean looks reasonable for all four models, both in the background and anomaly parts of the path. The mixture GPs have in common that one kernel models the background covariance and the other kernel models the anomaly regions where the observation values are fluctuating more. As a consequence, the resulting spatial correlation between locations is lower in the anomaly regions. When the kernels with shorter length scales are used for prediction, the predictive mean will follow the test data more closely. The single kernel model was trained on all the training observations, and the resulting length-scale parameter is a compromise between the spatial covariance in the background regions and the anomaly regions.

The length-scale parameter was constrained to be larger than 1.0 for all models. This constraint was set based on the density of observations – approximately every 0.5 m – to inform the training procedure that every observation must be correlated with the four nearest observations at the least. Without this constraint, the models for the anomaly regions would be overfitted to the training data.

Some overall remarks can be made about the performance of the four GP models shown in Fig. 3:

##### Smoothness

As expected, the NLE model exhibits nonlinearities where the background and anomaly regions meet. The reason is that which model is responsible for the prediction changes from one coordinate to the other. In comparison, the predictions from the MoE GP changes smoothly between background and anomaly regions. The purpose of the NLE-MA model is to smoothen the nonlinearities from the NLE GP by predicting an average of the background and anomaly models.

##### Variance

The predictive variance of the single kernel GP depends only on the spatial distance between observed locations and is more or less constant along the flight path. Close inspection of the NLE prediction shows that the variance is still depending only on the observation distance, but it is different in the background and anomaly regions. Finally, while the MoE GP predictive variance is similar to the single kernel models in the background regions, the variance in the anomaly region is generally higher than from the other models.

### Tjøtta Island

Figure 1 shows observations of methane concentration in the air above a farm on Tjøtta Island. A suspected source of methane flux was located near the centre of the survey area. Most of the observations in the training data set, and all of the observations in the test data set, were taken while the UAV followed a pre-planned grid path from the north-west to the south-east corner. The grid was oriented so that the flight lines were approximately 45$^{\circ }$ on the wind direction. This can be regarded as a compromise between dense sampling downwind and increasing the possibility to pass through the plume. Wind direction was predominantly from the north-east, which together with the location of the source was likely to influence where relatively high concentrations of gas would be observed. To ensure that downwind spatial dynamics was captured, a separate flight was done from the south-west side of the survey area and against the wind towards the suspected source of gas influx near the centre. Both the training and test observations indicate that there are generally higher concentrations of methane in the third quadrant of the survey area. This is coherent with a source being located near the centre of the area, and that mass transport of gas aligns with the downwind direction.

**Fig. 4:**
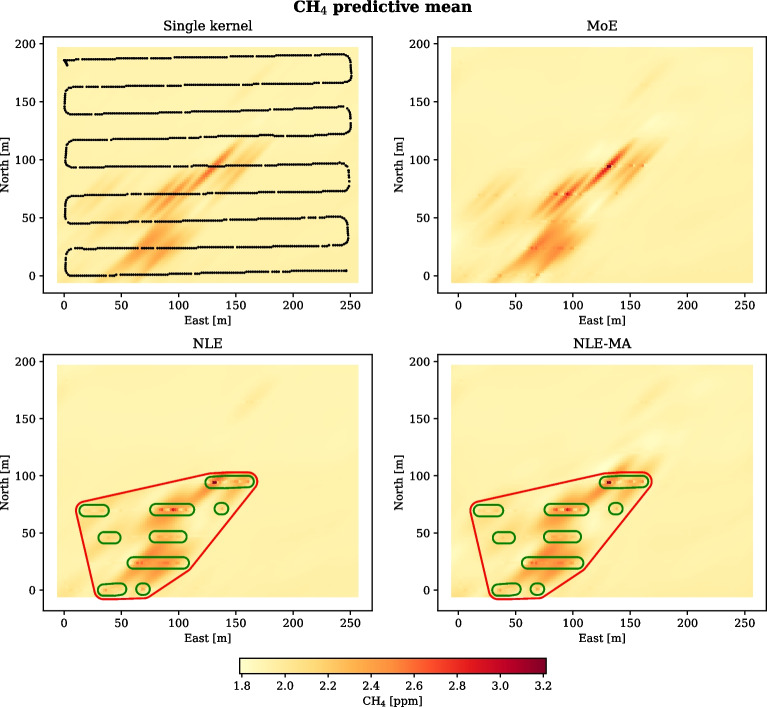
Predicted methane concentrations from the MoE, NLE, NLE-MA, and single kernel methods. In the plot showing predictions from the single kernel (upper right), black markers represent the locations of the test observations used to inform the predictions. In the other plots, observations are shown in a colour corresponding to the measured concentration. For the NLE and NLE-MA methods, the clusters that were classified as anomalously high are shown in green. Based on these clusters, an anomaly region was computed by Algorithm 1, shown in red

**Fig. 5:**
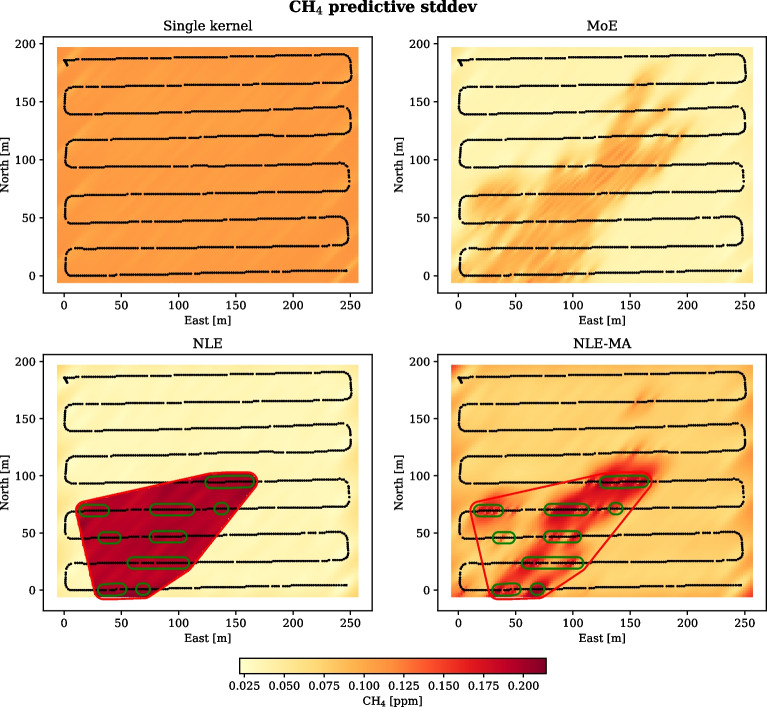
Predictive standard deviations for the MoE, NLE, NLE-MA, and single kernel methods. Black markers show the locations of the test observations that the predictions are based on. For the NLE and NLE-MA methods, the clusters and region that were classified as anomalously high are shown in green and red

#### MoE in 2D

Similar to the 1D setting with the data from Barter Island, the MoE GP was trained with the EM algorithm. The 10% of the observations with the highest methane concentrations in the training data set was used to initialize the anomaly model, while the remaining observations were assigned to the background model.

In order to use the MoE model for prediction, the final responsibilities for each sample were used to weigh kernel predictions at unsampled locations. To extend the responsibilities to the entire survey area, a gating GP was trained on the responsibilities for the test data. Then, predictions were made using each kernel and combined according to the gating GP.

Predictive means and standard deviations are shown in Figs. 4 and 5 (top left). The mean predictions are well aligned with the observations, although near observations with very high concentration the predictions are relatively low. High standard deviations coincide with locations where the two kernels give very different estimates. Most of the high deviations are found near the centre of the area and in the third quadrant, downwind of the probable emission source. Inspection of Fig. 5 (upper left) shows that the standard deviations are relatively low near observations and higher between the grid lines. This is particularly visible upwind and downwind of the high concentration measurements near the centre of the survey area. A quantitative assessment of the prediction performance is given in Table 2.

**Table 2:** Quantitative prediction performance of the different models with the data sets from Tjøtta

	RMSE	$\textrm{EIC}_{95}$	CRPS
MoE	**0.032**	0.994	**0.015**
NLE	0.048	0.997	0.018
NLE-MA	0.049	0.998	0.024
Single$^{1}$	0.047	**0.992**	0.031

$^{1}$A single kernel GP as presented in Eqs. 2–4

#### NLE and NLE-MA in 2D

Based on a given concentration threshold of 2.1 ppm, Algorithm 1 classified a subset of the measurements as being anomalously high and clustered the related locations first along the flight lines and then in 2D regions, as shown in Fig. 1 (left). Clusters of along-flight sample locations where the measured level suggests that an anomaly is present are shown in green. Based on the separation distance, the clusters were merged into two regions, shown in red, where the northernmost region consists of a single cluster of samples.

Two GP models were trained on the observations from the anomalous regions and on the observations of background concentrations, respectively. The trained models were then used with the observations from the test survey to predict the concentration at unsampled locations, shown in Figs. 4 and 5 (bottom). Algorithm 1 was run again on the test observations to identify regions where the kernel trained on anomalously high concentrations should be applied, encircled in red for the NLE and NLE-MA methods.

For the NLE method, predictions outside the region encircled in red are done with the kernel trained on background concentrations. These correspond well with the observations. Inside the anomaly region, the average predictions are markedly higher than outside, but not as high as the observed concentrations. The reason for this can be understood through considering the geometry of the anomaly region and the spatial distribution of anomalous observations: a challenge with employing the NLE method and Algorithm 1 in a 2D setting is that if the geometry of the region is such that a large part of the observations inside the region *do not* represent an emission, both training and prediction performance will approach that of a single kernel model.

The standard deviation in the NLE model is very low outside the anomaly region, since the background model has trained on a homogeneous data set of low concentration observations. Inside the anomaly region the standard deviation is markedly higher. There is a sharp gradient along the border between the regions, as a result of the high variance inside the anomaly region and the low variance outside.

The predictive means and standard deviations from the NLE-MA method are a result of smoothing between the predictions from the background model and the anomaly model. The means are very similar to the NLE model, both inside and outside of the anomaly region, which indicates that the gating function weighs the models correctly. The predicted standard deviations are generally higher inside the anomaly region than outside. However, the advantage of gating the model predictions based on likelihood is shown in that the map of standard deviation estimates is very detailed, even inside the anomaly region.

The prediction error, EIC, and CRPS as defined in Eqs. 15–17 are shown in Table 2 for each of the GP models.

## Discussion

The main difference between the GP modelling approaches that have been demonstrated here is that the model identification is either data-driven or knowledge-driven. For the MoE model, which observations should contribute to which mixture components—i.e. the clustering of data—is purely data-driven. There is no need for prior information, since the clustering adapts to the dynamics in the data set. In contrast, the observations belonging to the different components of the NLE models are identified with a threshold based on prior knowledge. An advantage with the latter method is that the cluster classification immediately reflects domain knowledge such as the natural background concentration of gases, or thresholds given in environmental legislation. In the case at Barter Island, the threshold was set based on a pre-survey and sensor performance, while for Tjøtta, the threshold was set based on the global trend of atmospheric methane and adding 10%. For both cases presented here, the difference between data-driven and knowledge-driven data clustering is relatively small. This confirms that the data-driven training procedure has resulted in mixture models that reflect the physical properties of the data set, i.e. one of the models is mainly responsible for observations where the gas concentration is above a certain value and the other model is responsible for the remaining observations.

Regarding feasibility of online usage, in the field during a survey, it is clear from Table 3 that the training procedure is more resource-demanding for the data-driven MoE than for the NLE methods. The difference between the NLE and NLE-MA methods can be ascribed to the training of the gating function. For any GP model, the size of the data set remains a challenge, due to the need to invert the correlation matrix to compute the likelihood. The processing power of embedded computers used in drones is usually limited compared to regular computers, and care should be taken to ensure that training can be completed successfully.

**Table 3:** Example CPU runtimes of the different models with the data sets from Tjøtta

	2D - Tjøtta	
	Training	Prediction
MoE	123	0.7
NLE	2.8	0.2
NLE-MA	25	0.3
Single$^{1}$	7.6	0.2

$^{1}$ A single kernel GP as presented in Eqs. 2–4

When it comes to prediction, a distinguishing feature between the MoE and the NLE-MA methods on one hand and the NLE method on the other hand is how they are applied across the survey area. In the former methods, predictions from the anomaly and background GPs are mixed relative to the location-specific likelihood of the two models. This mixing allows both models to contribute to predictions across the survey area. In contrast, the NLE method is predicting a location either using the anomaly model or the background model. As seen in Fig. 4, if the borders of the anomaly region are enclosing a high number of background concentration observations, the mean predictions of the anomaly GP will be lower than desired. This shortcoming is inherited by the NLE-MA method.

An advantage with the concept of spatial regions is that it gives us a way to model the directional anisotropy that can be introduced by wind or water currents. Figure 4, lower left, shows an example of directional anisotropy modelling. Here, high concentration is measured near the centre of the survey area, and as a result of the definition of region borders, predictions south-west of the high concentration are done with the anomaly GP that is trained on high concentration samples. The concentration predictions in quadrant 3 are therefore somewhat higher in the NLE model than in the single kernel model. Predictions in quadrant 1, 2, and 4 are done with a GP that is trained on background concentrations. Therefore, background level concentrations are predicted north and east of the area centre, even at locations that are relatively near high concentrations. These predictions are somewhat lower than predictions from the single kernel.

Location classification as done by Algorithm 1 should ideally capture gas dispersion downwind of a source, as shown in Fig. 4 (bottom). However, the shape of the anomaly region(s) is largely determined by the values of the input parameters to the algorithm, which puts the responsibility on users to define values for each scenario.

Adaptive sampling aims to help autonomous vehicles to accommodate situations and changes in the environment that were not foreseen when the survey was planned, and may therefore increase the chance that the gathered observations meet the survey goals. Surveys related to environmental monitoring of gas emission usually have several goals: area coverage, detection of emission, source localization, and emission quantification. Whether an emission source is present in the survey area may be unknown prior to the survey. In such cases, balancing the coverage goal with the goals that relate to the concentration values may increase the utility of the survey. Therefore, it is of particular interest to evaluate the belief state modelling approaches we have demonstrated here as to how they can be used for adaptive behaviour—whether the onboard navigation system can use the belief state to identify beneficial locations to sample in order to meet survey objectives. For instance, the area marked with a blue question mark upwind of the highest concentration in Fig. 1 (right) is interesting to sample in order to determine the location of the emission source more closely. The downwind area indicated with black question marks is interesting to sample to quantify the extent of the gas plume and overall emission. When the belief state is based on GP models, both the predictions themselves and the prediction uncertainty are quantified and available for use with adaptive sampling algorithms, so we therefore evaluate both aspects for the mixture methods in the following section. The predicted concentration and the uncertainties are shown in Figs. 4 and 5.

### Predictive accuracy

The accuracy of predicted concentrations is important if the goal of the mission is to provide detailed information about regions affected by emissions, e.g. locations where the concentration is likely to be above a set threshold. Such locations are often called excursion sets in literature, and examples of usage in adaptive sampling are given by for instance (Fossum et al., 2021) and Berget et al. (2023).

Figure 4 shows the predictive mean from the MoE, NLE, and NLE-MA models, along with predictions from a single kernel GP model as baseline. Common for all models is that they capture the anisotropy in the observations by exhibiting a diagonal pattern in the predictive means that is aligned with the wind direction. The MoE and single kernel models predict equally high concentrations upwind and downwind of the high concentration observation near the middle of the survey area. Downwind, the high prediction is coherent with mass transport of the gas. Upwind, the accuracy of the high concentration estimate depends on the actual location of the emission source, which is likely to be somewhere between the two grid lines at $\mathtt {\sim }100$ and $\mathtt {\sim }125$ m north. This area is marked with a blue question mark in Fig. 1, and taking observations in this area will help meet the subgoal of source localization. If the adaptive sampling is guided by an objective function to sample in areas with estimated high concentration, the areas upwind and downwind of the high concentration observations will be prioritized equally if the MoE or single kernel is used to model the belief state. In fact, the predictive mean of these methods will always encourage the vehicle to sample both upwind and downwind of high gas concentration observations.

Owing to the shape of the anomaly region, shown in red in Fig. 4 (bottom), the NLE methods estimate closer to background concentrations levels upwind of the highest concentration observations. An exploitative objective function based on an NLE method would keep the vehicle downwind of the highest concentration measurement and likely source. Compared to the MoE and single kernel methods, the vehicle would spend more survey time inside the anomaly region.

The NLE-MA predictions smoothen the background and anomaly models and do not exhibit the sharp gradients at the region border. Otherwise, the NLE-MA predictions are qualitatively identical to the NLE predictions.

Table 2 shows the RMSE between the observed values and the predicted means from the MoE, NLE, NLE-MA, and a single kernel GP. The MoE GP predictions are most aligned with the observations, with an RMSE that is 66% of the error from the other three models. We note that the error from the NLE and NLE-MA models are similar to the single kernel model. The similarity is likely to be due to the heterogeneity of observations values within the anomaly region, shown in Fig. 1 (left). Since the anomaly component was trained on samples both below and above the threshold value, the fitting of hyperparameters suffered. The EM algorithm used to train the MoE components handles data hetereogeneities by adding noise to the observations instead, which seems to make the MoE model better at predicting the true concentration value.

### Predictive uncertainty

An important feature of GP models is that predictions are probabilistic; each predicted mean of gas concentration at an unsampled location has an accompanying variance, indicating how certain the prediction is. An objective function that incorporates the model variance can reduce the uncertainty in the model by prioritizing to sample in areas with relatively high variance as demonstrated by Hitz et al. (2017). In practical applications, information about the uncertainty of a prediction is crucial for determining where additional measurements should be taken.

Figure 5 shows the standard deviations accompanying the concentration predictions based on the data set from Tjøtta. The standard deviations are computed by the kernel covariance functions, with the kernel parameters that were identified during the training procedure. Therefore, even though the structure of the kernel functions are the same, the parameters are different for the different models.

The single kernel model (top left) has only one set of parameters, which has to compromise between fitting the dynamics of the background and the anomalous observations. Therefore, the length scales identified through training are shorter than the length scales found for the models where one set of parameters could describe background concentrations only. As a result, the predicted standard deviations are near the middle of the scale. An objective function designed to reduce uncertainty in this map would prioritize unsampled locations based on the anisotropic distance to sampled locations.

In contrast, it is evident from Fig. 5 that the MoE, NLE, and NLE-MA models give relatively high standard deviation in areas near anomalous observations and lower standard deviations in areas with only background observations. This feature is desirable for online path planning, since it allows for prioritizing unsampled locations based on the uncertainty arising from the sampled concentration values in addition to the distance to locations that have already been sampled.

In the plot of the MoE predictions, Fig. 5 (top right), the anisotropic dynamics from wind dispersion is clearly visible along the north-east to south-west direction. The contribution from the anomaly component is weighted more in areas where the likelihoods of the observations are relatively high. Therefore, the resulting standard deviations are higher near observations that are above the background level. There is a correspondence between the estimates from the MoE and NLE-MA models in that the high variance is mainly ascribed to locations upwind and downwind of anomalous measurements, but the MoE has a generally lower standard deviation.

The NLE predictions in Fig. 5 (bottom left) come from two models trained on different subsets of the observations. This results in a background component with very small standard deviations, since all observations were below a given threshold. The anomaly component was trained on observations from the areas found by applying 1, which may contain a percentage of background observations along with the anomalous observations. Here, roughly half of the observations inside the area were below the anomaly threshold, which results in an anomaly component with high variance. While it is a simple extension of the single kernel predictions, basing an objective function on the NLE variance makes sense if the environmental forces are taken into account: Gas emission is likely to be patchy in a noisy environment, and emission is expected to be found downwind of the emission source. An objective function seeking to decrease uncertainty would stay inside the anomaly area.

The NLE-MA model weighs the predictions from the anomaly and background components based on the corresponding likelihood of nearby observations. Therefore, it can model patches of background concentration within the anomaly region, as well as high concentrations outside of it. In Fig. 5 (bottom right), the latter is visible as a patch of high variance near coordinates [160, 160]. Sampled concentrations in this area were higher than the usual background concentrations, but slightly below the anomaly threshold. The same region is visible in the plot of the MoE standard deviations. While overall the pattern of high standard deviations are similar in the MoE and NLE-MA predictions, the NLE-MA deviations are generally higher because they are partly based on the high standard deviations from the NLE anomaly component.

Table 2 shows the EIC and CRPS metrics for the different models. The EIC scores indicate that all models are conservative in that they estimate a higher uncertainty than is present in the data. This can be ascribed to differences in the training and test observations and the challenge of obtaining representative observations in a noisy environment. The CRPS takes into account the correctness of both the predicted means and the associated uncertainties and shows that the MoE model is best at estimating the true concentration with a realistic uncertainty. The NLE and NLE-MA models score better than the single kernel model, indicating that the application of two GP components enable the model to represent the uncertainty more truthfully than a single kernel model is able to.

Overall, a belief state based on the prediction uncertainty of the MoE and NLE-MA kernels will enable exploration upstream and downstream of the locations where high concentration was observed. An NLE-based belief state enables exploration in the anomaly region identified by Algorithm 1. In an environment with advective forces, this area will normally be downwind of the source(s).

## Conclusion

The main objective of this work was to evaluate the performance of different approaches to mixture GPs for autonomous robotic environmental monitoring. We have studied the advantages and challenges with data-driven vs. knowledge-driven data clustering for training and prediction with mixture models. The performance of mixture GPs for monitoring gas emissions has previously been demonstrated in controlled environments, and we extended this work to compare different methods of data clustering and mixture GPs on data sets acquired over large areas in windy environments.

In particular, we have demonstrated the application of three different mixture models on two different data sets and compared their predictive performance. All three mixture models have the advantage over the single kernel model that they allow the adaptive sampling to prioritize areas where high concentrations were observed, and avoid spending time in areas where the concentration is likely to be near background levels.

The MoE and NLE-MA models provide a map of prediction variance that is more nuanced than the map from the NLE model, in that it ascribes high variance based on discrepancy between models and not only distance from already sampled locations. This means that the prediction variance can be used for online path planning to prioritize new sampling locations upstream and downstream of locations where high concentration has already been observed. The ability to do this prioritization enables more cost-effective localization and quantification of emissions, in turn achieving goals of environmental monitoring surveys.

In addition to providing nuanced prediction variances, the MoE model predicts both background and anomalous emissions with smaller error and more correct variances than the NLE models and is therefore both the most flexible and accurate model. It is worth to note that the data-driven training procedure of the MoE method results in mixture components of anomaly and background concentrations that correspond to the physical properties of the data sets. The data sets resulting from the knowledge-driven hard clustering of regions for NLE models are likely to contain a mix of background and anomaly measurements. However, the NLE and NLE-MA models are faster to train than the MoE model, making them more suited for online path planning on devices with limited processing power.

While progress is continuously being made towards practical adaptive sampling algorithms for environmental monitoring, their successful application is likely to depend on how well we understand the environment. As seen in the data sets presented herein, gas emission plumes may be intermittent, or patchy, in nature. The drivers behind the patchiness include the nature of the emission itself, which can be a steady influx or come in bursts, variation in the forces that drive mass transport such as the speed and direction of the wind, and finally the dispersion and buoyancy of the emitted gas. The MoE method is more flexible and accurate and will likely be the first choice for belief state modelling in such environments. However, if the online processing power of autonomous vehicles struggle to accommodate the data-driven training procedure, our results show that NLE and NLE-MA models can be employed. Especially, the NLE-MA model can model a belief state with upwind and downwind uncertainties similar to the MoE model, with a CRPS fairly close the MoE model. If the monitoring scenario is well known, and the mass transport is fairly stable, the knowledge-driven NLE and NLE-MA methods may be preferred because they efficiently establish the anomaly regions to focus the adaptive sampling on.

The natural next step to further mature these methods is application in an online learning and path planning context, where the belief state is continuously updated with newly acquired samples and the next location to sample is computed based on the belief state.

## Data Availability

Dataset 1 is available as specified in the manuscript, or at https://doi.pangaea.de/10.1594/PANGAEA.898636. Dataset 2 is available as specified in the manuscript or at https://zenodo.org/records/13691531.

## Code availability

The code used to create the results presented here is available under the Creative Commons licence at https://github.com/ivarkristian/mixture_gps.
